# cART Exacerbates Cocaine-Induced Cortical Neuron Hyperactivity in Non-Transgenic but Not HIV-1 Transgenic Rats

**DOI:** 10.3390/membranes16040115

**Published:** 2026-03-27

**Authors:** Tabita Kreko-Pierce, Lihua Chen, Guojie Qu, Stefanie L. Cassoday, Lena Al-Harthi, Xiu-Ti Hu

**Affiliations:** Department of Microbial Pathogens & Immunity, RUSH University Medical Center, Chicago, IL 60612, USA; tabita_kreko-pierce@rush.edu (T.K.-P.); lihua_chen@rush.edu (L.C.); stefanie_l_cassoday@rush.edu (S.L.C.); lena_al-harthi@rush.edu (L.A.-H.)

**Keywords:** neuroHIV, medial prefrontal cortex, cocaine, combination antiretroviral therapy, neuronal excitability, electrophysiology

## Abstract

HIV-associated neurocognitive disorders (HAND) persist despite combination antiretroviral therapy (cART) and can be exacerbated by repeated cocaine (COC) exposure. Because COC, HAND, and cART independently disrupt medial prefrontal cortex (mPFC) function, their combined neurotoxic impact is a critical clinical concern. Using patch-clamp electrophysiology in HIV-1 transgenic (Tg) and non-Tg rats, we examined mPFC pyramidal neuron activity following repeated exposure to COC and/or cART. In non-Tg rats, COC and cART independently increased neuronal firing, trending toward an additive hyperactive effect when combined. Conversely, HIV-1 Tg rat neurons exhibited plateaued excitability, with no further firing elevations induced by COC or cART. Under intense depolarizing stimuli, treated neurons displayed overactivation-induced firing declines. These findings indicate that while COC and cART additively disrupt mPFC function in non-Tg rats, excitability mechanisms appear saturated in the HIV-1 Tg model. This restricted experimental context highlights the overlapping neurobiological impacts of cART and stimulant use, providing foundational insights into the comorbidity of COC use disorder and HAND.

## 1. Introduction

Despite combination antiretroviral therapy (cART) advances, HIV-1 remains associated with HIV-associated neurocognitive disorders (HAND) [1,2,3]. HAND and the underlying viral protein-induced neurotoxicity in the central nervous system are collectively referred to as neuroHIV [1,4]. While cART reduces viral load and improves survival, cognitive and behavioral deficits persist, often tied to mPFC dysfunction [5,6,7,8,9,10]. Chronic cART, particularly Triumeq, abnormally increases mPFC excitability [11], raising concerns about its potential role in HAND.

COC use disorder is a common comorbidity of HIV/AIDS, inducing mPFC neuronal dysfunction that could worsen HAND [9,10,11,12]. In combination with neuroHIV-like conditions [13], COC further disrupts cortical neuron activity [14,15]. While cART suppresses HIV replication, its long-term impact on brain function remains unknown, as both viral proteins and antiretrovirals may induce neuronal injury [5,6].

In the present study, we examined how daily repeated COC exposure alters mPFC neuron activity induced by HIV-1 proteins in both non-transgenic (non-Tg) control rats and the HIV-1 Tg rat model [13]. The HIV-1 Tg model was specifically chosen because it constitutively expresses neurotoxic HIV-1 viral proteins (such as Tat and gp120) without active viral replication, making it an excellent and widely accepted model for studying the pathophysiology of neuroHIV in the modern cART era [6,16,17,18,19,20,21]. Using a patch-clamp approach, we assessed whether COC and/or cART exacerbate viral protein-induced mPFC neuron hyperactivity, aiming to inform potential strategies to mitigate neuroHIV complicated by substance use.

## 2. Materials and Methods

### 2.1. Animals

**Figure 1:**
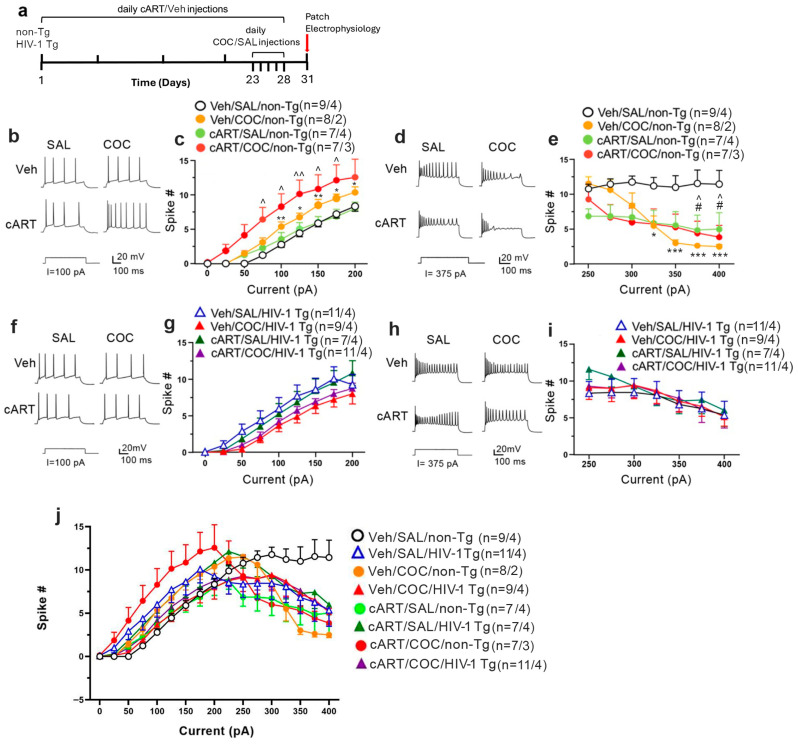
Additive effects of repeated cocaine (COC) and cART on mPFC neuronal hyperactivity in non-Tg rats are absent in HIV-1 Tg rats. (**a**) Schematic of the experimental design. Rats received daily s.c. injections of cART or vehicle (Veh) for 28 days. From days 23 to 28, rats received additional daily s.c. injections of COC or saline (SAL). Following a 3-day withdrawal, rats were sacrificed for patch-clamp recordings. (**b**) Representative evoked firing traces in mPFC neurons from SAL- vs. COC-pretreated non-Tg rats, with or without cART. (**c**) Current-spike response curves displaying a significant increase in firing of mPFC neurons from COC- and cART-treated non-Tg rats compared to Veh/SAL controls, with a trend toward an additive effect in the cART/COC/non-Tg group. (**d**) Representative traces showing overactivation-induced decline under high stimuli (>200 pA) in non-Tg rats. (**e**) Current-spike curves detailing the significant decrease in high-stimuli firing in treated non-Tg rats. (**f**) Representative evoked firing traces in HIV-1 Tg rats. (**g**) Current-spike response curves in HIV-1 Tg rats demonstrating a lack of significant additive hyperactivity following COC or cART treatment. (**h**) Representative traces under high stimuli in HIV-1 Tg rats. (**i**) Current-spike curves for high stimuli in HIV-1 Tg rats, showing no significant differences between treatment groups. (**j**) Composite plot of spike frequencies comparing the overall treatment patterns between non-Tg and HIV-1 Tg rats. Data are mean ± SEM. *, **, *** *p* < 0.05, 0.01, 0.001: Veh/SAL/non-Tg vs. Veh/COC/non-Tg; # *p* < 0.05: Veh/SAL/non-Tg vs. cART/SAL/non-Tg; ^, ^^ *p* < 0.05, 0.01: cART/COC/non-Tg vs. Veh/SAL/non-Tg.

6-month-old male HIV-1 Tg and non-Tg rats (Envigo, Indianapolis, IN, USA) were housed at Rush University Animal Facility. All procedures were approved by the Rush University Medical Center IACUC (protocol code 23-050 and date of approval 20 October 2023) and conducted according to NIH and institutional guidelines. Rats were group-housed (two/cage) on 12 h light/dark cycles with ad libitum access to food and water. Rats received daily subcutaneous (s.c.) injections of Triumeq (cART; ABC 12 mg/kg, DTG 1 mg/kg, and 3TC 6 mg/kg) or vehicle (veh, 20% DMSO) for 28 consecutive days, a regimen previously established to model cART effects in rats [11]. During the final 5 days of cART treatment (Days 23–28), animals also received once-daily s.c. injections of cocaine (COC; 15 mg/kg/day) or saline (SAL; 0.1 mL/kg/day) [22,23]. This repeated, non-contingent COC exposure was followed by a 3-day drug-free withdrawal period prior to electrophysiological recordings [24] (Figure 1a). Sample sizes for each group were as follows: Veh/SAL/non-Tg: 4 rats (9 neurons), Veh/COC/non-Tg: 2 rats (8 neurons), cART/SAL/non-Tg: 4 rats (7 neurons), cART/COC/non-Tg: 3 rats (7 neurons); Veh/SAL/HIV-1 Tg: 4 rats (11 neurons), Veh/COC/HIV-1 Tg: 4 rats (9 neurons), cART/SAL/HIV-1 Tg: 4 rats (7 neurons), cART/COC/HIV-1 Tg: 4 rats (11 neurons).

### 2.2. Slice Preparation

Rats were deeply anesthetized with isoflurane and transcardially perfused with ice-cold, oxygenated cutting solution. The cortex was dissected, placed in the same solution for two minutes, and sectioned into 300 µm coronal mPFC slices using a vibratome (VT1000S, Leica Biosystems, Nussloch, Germany). Slices were transferred to a recovery chamber with artificial cerebrospinal fluid (aCSF) oxygenated with carbogen (95% O_2_/5% CO_2_) and incubated at room temperature for 1 h before assessment [25].

### 2.3. Electrophysiology

Slices were superfused with aCSF (~2 mL/min) at room temperature. mPFC pyramidal neurons (layers V–VI) were visualized via DIC microscopy (40× water-immersion, Eclipse E600FN, Nikon Corporation, Tokyo, Japan). Patch electrodes (4–6 MΩ, borosilicate glass, Warner Instruments, Hamden, CT, USA) containing an internal solution (mM: 120 K-gluconate, 10 HEPES, etc.) were used to assess neuron’s firing [11,13,26]. Action potentials (APs) were evoked by depolarizing pulses (500 ms, 0–400 pA, 25 pA increments). Neurons met the criteria if the resting membrane potential was more hyperpolarized than −60 mV and the AP amplitude exceeded 60 mV (for vehicle/SAL-pretreated non-Tg) or 50 mV (for other groups) for the first evoked AP. APs with an amplitude < 30 mV were excluded from analysis. The slightly lower 50 mV threshold for transgenic group was used to avoid artifactually excluding the expected pathological neuronal phenotypes, as chronic viral protein inherently alters membrane properties, resulting in natively smaller APs.

### 2.4. Statistics

Spike data were analyzed using a two-way repeated-measures analysis of variance (ANOVA), with current as the repeated factor, followed by Sidak’s multiple comparison post hoc test. In these analyses, individual neurons were treated as the independent statistical unit. Outlier exclusion rules (≥2 standard deviations from the mean or not meeting criteria) were established and applied symmetrically and blindly across all groups. Specifically, one cell was excluded from the Veh/SAL/non-Tg group and one from the cART/COC/HIV-1 Tg group. Data are presented as mean ± SEM, with statistical significance set at *p* < 0.05.

## 3. Results

Repeated COC exposure significantly increased firing of mPFC pyramidal neurons in non-Tg rats compared to SAL-pretreated controls (Figure 1b,c, Veh/SAL/non-Tg vs. Veh/COC/non-Tg: treatment: F_(1,15)_ = 8.939, *p* = 0.0092; current × treatment: F_(8,120)_ = 4.775, *p* < 0.0001). As expected, based on our prior findings [11], these results indicate that cART can independently increase neuronal excitability. Consequently, non-Tg rats that received the combined cART and COC treatment exhibited robust, significantly elevated firing compared to baseline vehicle/saline controls (Veh/SAL/non-Tg vs. cART/COC/non-Tg: treatment: F_(1,14)_ = 7.861, *p* = 0.0141; current × treatment: F_(8,112)_ = 4.532, *p* < 0.0001). Furthermore, this combined exposure trended toward an additive effect, enhancing the hyperactivity beyond that of cART alone (cART/SAL/non-Tg vs. cART/COC/non-Tg: treatment: F_(1,12)_ = 4.595, *p* = 0.0533; current × treatment: F_(8,96)_ = 2.998, *p* = 0.0048). These findings indicate that cART can independently increase neuronal excitability in the absence of HIV-1 protein expression [11].

HIV-1 Tg rats exhibited a trend towards elevated basal firing relative to Veh/SAL/non-Tg controls (Figure 1e,f, Veh/SAL/non-Tg vs. Veh/SAL/HIV-1 Tg: treatment: F_(1,18)_ = 3.338, *p* = 0.0843; current × treatment: F_(8,144)_ = 2.227, *p* = 0.0290), consistent with previous reports [11,13]. However, post hoc analysis did not confirm a robust main effect across all currents, suggesting that the effect may be collapsed across treatment conditions. Notably, COC failed to further increase neuronal firing in HIV-1 Tg rats (Figure 1f,g, Veh/SAL/HIV-1 Tg vs. Veh/COC/HIV-1 Tg: treatment: F_(1,18)_ = 2.215, *p* = 0.1540; current × treatment: F_(8,144)_ = 1.111, *p* = 0.3594), diverging from earlier studies where self-administered COC and HIV-1 proteins synergistically elevated mPFC excitability [11,13]. Similarly, cART did not significantly augment firing in HIV-1 Tg rats (Veh/COC/HIV-1 Tg vs. cART/COC/HIV-1 Tg, F_(1,18)_ = 0.5738, *p* = 0.4586; current × treatment: F_(8,144)_ = 0.5738, *p* = 0.4586). This may reflect shared mechanisms of excitability that are already saturated, or possibly a modulatory action of Triumeq that prevents further increases in neuronal activity. Collectively, these results demonstrate a unified pattern: while repeated COC and cART additively increase mPFC neuron firing in non-Tg rats, this additive effect is absent in HIV-1 Tg rats, suggesting a plateau in excitability.

Under low-intensity depolarizing stimuli (≤200 pA), mPFC neurons from HIV-1 Tg rats exhibited a trend toward decreased firing compared to non-Tg controls under both vehicle/saline and COC-treated conditions (Figure 1c,g; Veh/SAL/HIV-1 Tg vs. Veh/SAL/non-Tg: F_(1,18)_ = 3.338, *p* = 0.0843; current × treatment: F_(8,144)_ = 2.223, *p* = 0.0290; Veh/COC/HIV-1 Tg vs. Veh/COC/non-Tg: F_(1,15)_ = 3.214, *p* = 0.0932; current × treatment: F_(8,120)_ = 1.873, *p* = 0.0703). While cART treatment alone did not significantly alter firing in HIV-1 Tg rats relative to cART-treated non-Tg controls (Figure 1c,g; cART/SAL/HIV-1 Tg vs. cART/SAL/non-Tg: F_(1,12)_ = 1.451, *p* = 0.2515; current × treatment: F_(8,96)_ = 1.689, *p* = 0.1110), the combined exposure to cART and COC significantly decreased firing in HIV-1 Tg rats compared to identically treated non-Tg animals (cART/COC/HIV-1 Tg vs. cART/COC/non-Tg: treatment: F_(1,16)_ = 6.216, *p* = 0.0240; current × treatment: F_(8,128)_ = 2.714, *p* = 0.0086).

Under high-intensity depolarizing stimuli (>200 pA), mPFC neurons displayed a current-associated firing reduction indicative of overactivation-induced firing decline (Figure 1d,e,h–j). In non-Tg rats (Figure 1d,e,j), both COC and cART individually decreased firing relative to Veh/SAL controls, suggesting compensatory mechanisms to excessive excitation controls (non-Tg: Veh/SAL/non-Tg vs. Veh/COC/non-Tg: treatment: F_(1,15)_ = 12.80, *p* = 0.0027; current × treatment: F_(6,90)_ = 9.447, *p* < 0.0001; Veh/SAL/non-Tg vs. cART/SAL/non-Tg: treatment: F_(1,14)_ = 8.088, *p* = 0.0130; current × treatment: F_(6,84)_ = 1.730, *p* = 0.1240). Moreover, cART-treated non-Tg rats that also received COC exhibited further reduction in firing (Veh/SAL/non-Tg vs. cART/COC/non-Tg: treatment: F_(1,14)_ = 8.088, *p* = 0.0130; current × treatment: F_(6,84)_ = 1.730, *p* = 0.1240).

Under high-intensity depolarizing stimuli (>200 pA), HIV-1 Tg rats showed a trend toward greater overactivation-induced firing decline compared to non-Tg controls (Figure 1e,i, Veh/SAL/HIV-1 Tg vs. Veh/SAL/non-Tg: F_(1,18)_ = 3.315, *p* = 0.0853; current × treatment: F_(6,108)_ = 1.145, *p* = 0.3414). However, neither COC nor cART alone significantly reduced firing in HIV-1 Tg rats relative to SAL controls, and COC failed to further reduce firing in cART-treated HIV-1 Tg animals (Veh/SAL/HIV-1 Tg vs. Veh/COC/ HIV-1 Tg: treatment: F_(1,18)_ = 0.04408, *p* = 0.8361; current × treatment: F_(6,108)_ = 0.09689, *p* = 0.9966; Veh/SAL/HIV-1 Tg vs. cART/SAL/ HIV-1 Tg: treatment: F_(1,16)_ = 0.2519, *p* = 0.6226; current × treatment: F_(6,96)_ = 0.6533, *p* = 0.6874; Veh/SAL/HIV-1 Tg vs. cART/COC/HIV-1 Tg: treatment: F_(1,20)_ = 0.04956, *p* = 0.8261; current × treatment: F_(6,120)_ = 0.0.1358, *p* = 0.9914). This lack of effect further supports the concept of a limited dynamic range for excitability modulation in the HIV-1 Tg model.

## 4. Discussion

The present study reveals that repeated exposure to COC increases mPFC neuronal excitability in non-Tg rats in response to low-intensity depolarizing stimuli (≤200 pA). Furthermore, cART trended toward potentiating this COC-induced neuronal dysfunction. These results align with prior findings demonstrating that repeated COC exposure enhances calcium influx and disrupts potassium channel function in the mPFC [26], and that cART—particularly the Triumeq regimen—increases excitability via L-type voltage-gated calcium channels [11]. In HIV-1 Tg rats, mPFC neurons also exhibited a trend toward elevated baseline excitability under lower-intensity stimuli (≤200 pA) (Figure 1c,g; Veh/SAL/non-Tg vs. Veh/SAL/HIV-1 Tg). This is consistent with previous studies showing that HIV-1 viral proteins contribute to synaptic and excitability disturbances through mechanisms involving calcium dysregulation and altered glial–neuronal signaling [11,27,28,29,30,31]. Together, these data implicate COC, cART, and HIV-1 proteins as key contributors to the abnormal hyperexcitability of cortical neurons, raising concerns about their individual and combined neurotoxic potential.

Under conditions of stronger excitatory stimuli (>200 pA), mPFC neurons repeatedly exposed to COC, cART, or pre-existing HIV-1 proteins exhibited initial overactivation followed by a marked decline in firing—a dysregulated response not observed in Veh/SAL-treated non-Tg controls. Notably, neither COC nor cART produced any additional significant effects on excitability in HIV-1 Tg rats in response to stronger excitatory stimuli. This lack of an additional effect under strong depolarizing currents is consistent with our previous work showing that COC- and/or HIV-induced neuronal overactivation triggers a firing decline that is absent in healthy (SAL/non-Tg) controls. The absence of an additive effect suggests a convergent or saturable mechanism. One working hypothesis is that the chronic presence of HIV-1 proteins in the brain primes or exhausts the molecular pathways affected by COC and cART, resulting in a plateaued neuropathophysiological response. Alternatively, neuroplastic adaptations in HIV-1 Tg rats could buffer the neurons against additional excitatory insults.

The mechanisms underlying mPFC hyperexcitability observed in this study likely involve multiple interacting cellular pathways. Our previous work has shown that repeated cocaine exposure and chronic cocaine self-administration both aberrantly increase calcium influx through voltage-gated calcium channels and disrupt potassium channel function, contributing to heightened neuronal excitability in the mPFC [26]. In addition to these effects, dysregulation of L-type calcium channels by cART [11], along with enhanced excitatory glutamatergic signaling or diminished inhibitory GABAergic tone due to chronic cocaine exposure or neuroHIV, may further destabilize firing patterns in mPFC neurons. While these intrinsic and synaptic alterations likely contribute to the neuronal dysfunction observed under these experimental conditions, the current study did not include direct molecular readouts or cognitive assessments. Therefore, these mechanistic pathways and their direct link to HAND-like behavioral phenotypes remain working hypotheses derived from our prior in vivo models [11,26]. Future investigations must explicitly test these saturable mechanisms using targeted ion channel pharmacology and assess their broader neurobehavioral consequences.

Importantly, the current findings diverge from our previous studies using drug self-administration models, where repeated COC exposure significantly enhanced HIV-1 protein-induced mPFC excitability in response to lower excitatory stimuli (25–100 pA) [11,13]. This discrepancy may be attributed to the mode of cocaine delivery. Although both modes of administration lead to mPFC neuronal hyperactivity, non-contingent (experimenter-administered) exposure—as used in this study—lacks the behavioral reinforcement and motivational components inherent in self-administration paradigms. Contingent drug exposure has been shown to induce more robust neuroadaptations, including greater upregulation of dopamine transporter expression and altered synaptic plasticity [29,32,33]. Thus, the neurobiological consequences of cocaine exposure may differ significantly depending on the behavioral context in which the drug is taken.

The short (3-day) withdrawal period used in this study also warrants consideration. Our previous studies have demonstrated that mPFC neuronal hyperexcitability persists across both short- and long-term withdrawal phases, suggesting that cocaine-induced neuroadaptations can be long-lasting and independent of the drug’s presence [26,27]. Nevertheless, the specific trajectory and magnitude of these effects likely vary across time. Future studies should examine broader temporal windows to determine how excitability evolves from acute withdrawal to longer-term abstinence, particularly in the context of co-occurring cART or neuroHIV exposure.

We acknowledge several important limitations in the current study that warrant consideration. First, in our statistical approach, the neuron was treated as the independent statistical unit (n = 7–11 per group), drawn from a small sample size of animals (n = 2–4 rats per group). While this is a common and widely accepted practice in slice electrophysiology, we fully acknowledge that this violates the assumption of independence and risks inflating Type I error. Consequently, these findings should be viewed as preliminary trends appropriate for a short Communication, and future fully powered studies will employ linear mixed-effects models to appropriately account for nested data.

Second, the current experimental design utilized exclusively male subjects to match our historical baselines. Recognizing sex as a fundamental biological variable in the pathophysiology of neuroHIV and addiction, the exclusion of female cohorts limits the generalizability of our conclusions. It is imperative that future investigations assess the potential sex-specific effects of COC, cART, and their interplay. This direction is especially critical given our recent findings, which indicate that psychostimulant exposure, such as methamphetamine self-administration, drives mPFC neuronal dysfunction in a profoundly sex- and withdrawal time-dependent manner [34,35].

Lastly, as noted, our non-contingent cocaine administration model does not fully replicate the complex behavioral dynamics of human drug use. While our previous studies demonstrate that both self- and experimenter-administered COC induced mPFC neuronal hyperexcitability [26,35], contingent paradigms could produce stronger or qualitatively different neuronal adaptations. Additionally, the different withdrawal times (3d vs. 14–18d) could also contribute to less pronounced effects. These differences highlight the importance of dose and behavioral context in shaping drug-induced neural outcomes.

## 5. Conclusions

This study demonstrates that daily non-contingent exposure to COC and cART contributes to an abnormal increase in mPFC neuronal excitability in non-Tg rats. While COC and cART individually disrupt neuronal firing and trend toward additive effects in non-Tg animals, they do not further exacerbate neuronal dysfunction in HIV-1 Tg rats, suggesting a plateaued excitability state. These findings highlight the complex, non-linear interplay between drug exposure, antiretroviral treatment, and neuroHIV and underscore the importance of administration paradigms in shaping cortical neuron outcomes. Future works should investigate how motivation and reinforcement modulate these effects, explore longer withdrawal periods, and incorporate both sexes to improve translational relevance in understanding the neurobiological mechanisms underlying HAND and comorbid substance use.

## Data Availability

The data and statistical analysis supporting the findings of this study are publicly available in the GitHub repository at https://github.com/HulabRush/Coc-HIV-cART-firing-data-.git (accessed on 6 February 2026) [36].

## Abbreviations

The following abbreviations are used in this manuscript:

aCSF	Artificial cerebrospinal fluid
ANOVA	Analysis of variance
AP	Action potential
cART	combination antiretroviral therapy
COC	Cocaine
HAND	HIV-associated neurocognitive disorders
IACUC	Institutional Animal Care and Use Committee
mPFC	Medial prefrontal cortex
SAL	Saline
SEM	Standard error of the mean
Tg	Transgenic
Veh	Vehicle
